# Synovitis, Acne, Pustulosis, Hyperostosis, Osteitis (SAPHO) Syndrome: Clinical and Therapeutic Aspects

**DOI:** 10.7759/cureus.18332

**Published:** 2021-09-27

**Authors:** Kaba Condé, Carlos Othon Guelngar, Awada Mohamed, Emmanuel Adjibaye, Fodé Abass Cissé

**Affiliations:** 1 Department of Rheumatology, Ignace Deen National Hospital, University of Conakry, Conakry, GIN; 2 Department of Neurology, Ignace Deen National Hospital, University of Conakry, Conakry, GIN; 3 Department of Infectious Diseases, Reference Hospital, N’Djamena, TCD

**Keywords:** synovitis, acne, pustulosis, hyperostosis, sapho syndrome, conakry-guinea

## Abstract

Synovitis, acne, pustulosis, hyperostosis, osteitis (SAPHO) syndrome is a rare entity. It is frequently under-detected. We report the case of SAPHO syndrome in a 38-year-old woman, seen in consultation for pain and swelling of the anterior chest wall affecting the sternoclavicular and sternocostal joints predominantly on the right, and low back pain with an inflammatory appearance with peripheral damage, especially in the legs. We also found in our patient episodes of palmoplantar pustulosis. The diagnosis of SAPHO syndrome was retained in accordance with Kahn's diagnostic criteria, and the osteitis was confirmed by morphological examinations (CT scan, MRI, and bone scintigraphy). The patient was treated with non-steroidal anti-inflammatory drugs and methotrexate with good clinical improvement.

## Introduction

Synovitis, acne, pustulosis, hyperostosis, osteitis (SAPHO) syndrome is an autoinflammatory disease, which refers to the association of a heterogeneous set of cutaneous and osteoarticular manifestations [1]. In 1961, Windom et al. were the first to describe the joint involvement associated with acne conglobata [2]. It was not until 1987 that Chamot et al. combined these disparate entities under the acronym SAPHO [3]. The prevalence of SAPHO syndrome does not exceed 1/10,000 worldwide [4]. The disease is rare, often unrecognized, and characterized by significant inflammatory manifestations of the skin and joints [5,6]. The syndrome is sometimes incomplete and may overlap with other entities causing diagnostic confusion [7]. Here, we present the case of a 38-year-old woman and review the literature.

## Case presentation

**Figure 1:**
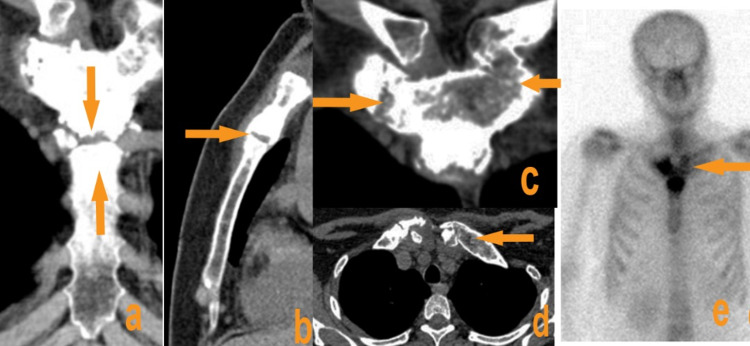
Chest CT scan (a) Coronal and (b) sagittal sections showing chronic osteitis of the manubriosternal joint with ankylosis (arrow). (c) Coronal section: erosion and hyperostosis of the costosternal joints (arrow). (d) Partial fusion of the left sternoclavicular joint (arrow). (e) Hyperfixation of the anterior chest wall on whole-body scintigraphy shows a bull's horn appearance.​​​​​​​

A 38-year-old woman with no particular history was seen in consultation for pain and swelling of the anterior chest wall (ACW) associated with the sternoclavicular and sternocostal joints predominantly on the right, the knees, and ankles, progressing with outbreaks interspersed with remission for around two years. In the history, episodes of palmoplantar pustulosis were noted. There was no concept of inflammatory rheumatism, nor tumor, or infection. On examination, the patient was in good general condition, with pain associated with swelling and sometimes redness of the sternoclavicular and sternocostal joints, more marked on the right. There was also synovitis of the knees and ankles. In the lumbar region, the patient reported pain on pressure of the spinous processes with stiffness (Schöber index 10 + 3). Pain on sacroiliac pressure with positive Ericksen's and Volkmann's maneuvers was also observed. Otherwise, the rest of the physical exam was normal. The laboratory workup showed a normal blood count, a non-specific biological inflammatory syndrome with an erythrocyte sedimentation rate (ESR) accelerated to 47 mm at the first hour, a positive C-reactive protein (CRP) at 12.6 mg/l. Immunologically, anti-nuclear antibodies (ANA), anti-citrullinated cyclic peptide antibodies (anti-CCP), rheumatoid factors (RF), and HLA-B27 antigen were negative. The chest CT scan (Figures 1a-1d) showed chronic osteitis in the sternal manubrium with involvement of the costosternal joint of the first ribs bilaterally. Bone scintigraphy (Figure 1e) showed the bull's horn sign in the thorax with more marked hyperfixation on the left.

**Figure 2:**
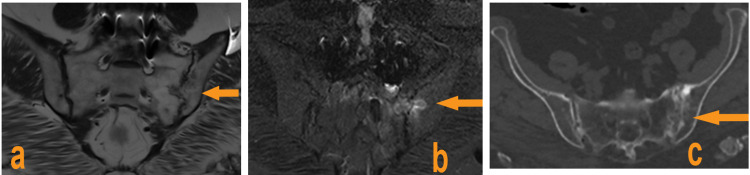
MRI sequence T1 (a), and T2 (b), and (c) CT scan of the sacroiliac joints showing sequelae sacroiliitis predominant on the left

There was a partial fusion of the distal part of the clavicle and the first rib on the left side. MRI and CT scan of the sacroiliacs (Figures 2a-2c) showed almost complete fusion of the bilateral sacroiliac joint space, especially on the left.

The diagnosis of SAPHO syndrome was made due to the involvement of anterior chest wall (ACW), the presence of synovitis, episodes of palmoplantar pustulosis, imaging, in accordance with the modified Kahn criteria. The patient was put on a treatment with naproxen 500 mg twice per day and methotrexate 15 mg per week, which showed good improvement.

## Discussion

SAPHO syndrome is a chronic immune-mediated condition that refers to the association of a heterogeneous set of cutaneous and osteoarticular manifestations [1]. The acronym of the syndrome reflects the varying combination of synovitis, acne, pustulosis, hyperostosis, and osteitis [1,2]. SAPHO is a rare disease, its prevalence is estimated <1/10,000, but it may be higher since it is a frequently underdiagnosed condition [4]. This disease affects both adults and children, although it is seen predominantly in women. As seen in our patient, the average age of getting the disease is estimated at 38 years [5,8]. The etiopathogenic mechanism of SAPHO syndrome remains poorly understood, although some authors have described hypotheses involving genetic, immunological, and bacteriological factors such as autophagy, interleukin-1, tumor necrosis factor (TNF), and *Propionibacterium* *acnes *[9,10]. SAPHO syndrome was initially classified as a spondyloarthritis [5,11]. However, in this syndrome, sacroiliitis is often unilateral and associated with hyperostosis [6,11]. 

There is no clear association with the HLA-B27 antigen, with a reported prevalence between 4% and 17% [7,11]. Indeed, our patient was HLA-B27 negative but presented with unilateral sacroiliitis. Recent data suggest that SAPHO syndrome belongs to primary inflammatory osteitis in the spectrum of autoinflammatory diseases [12,13]. The clinical presentation of SAPHO syndrome is heterogeneous and insidious. It is estimated that approximately 60%-95% of patients with possible SAPHO also suffer from anterior chest wall syndrome, which typically involves the sternum, collarbones, and/or sternoclavicular, manubriosternal joints in different combinations [6,11,14]. Axial involvement is present in 32%-52% of patients with SAPHO syndrome [6,14]. Skin involvement usually precedes the onset of joint symptoms; however, it can occur at any time during the disease [15]. The main skin abnormalities are palmoplantar pustulosis, pustular psoriasis, psoriasis vulgaris, severe acne, or suppurative hidradenitis [8,11]; our patient presented with episodes of palmoplantar pustulosis. Chamot et al. [3] proposed the initial diagnostic criteria for SAPHO syndrome in 1987, but Kahn et al's proposal in 1994 [16], modified in 2003, is the most frequently used. However, it is difficult to make the diagnosis in the absence of skin lesions [11]. Laboratory workup and imaging results play a crucial role. Some studies have shown that erythrocyte sedimentation rate (ESR) and C-reactive protein (CRP) can be elevated in SAPHO syndrome [17,18], and this has been confirmed by our case. Additionally, we did not detect any autoantibodies, including anti-nuclear antibodies (ANA), rheumatoid factors (RF), anti-citrullinated cyclic peptide antibodies (anti-CCP), and HLA-B27. Bone scintigraphy showed a characteristic image of the SAPHO syndrome's "bull horn aspect" at the level of the ACW [10]. As reported in our case, the chest CT scan showed chronic osteitis in the sternal manubrium with involvement of the costosternal joint. CT scan and MRI of the sacroiliac joints revealed a left unilateral sacroiliitis. Bone biopsy is only performed in atypical forms, in particular, to exclude a tumor [10].

To our knowledge, there is no effective treatment for SAPHO syndrome. The main focus of treatment is on relieving symptoms. Non-steroidal anti-inflammatory drugs (NSAIDs) are the first line of treatment [11]. Tetracyclines and macrolides are an alternative treatment, given the possible role of *P.* *acnes *in the pathogenesis of the disease. However, their effectiveness is lost with weaning [7]. Several studies have confirmed the effectiveness of bisphosphonates [19], steroids, methotrexate, anti-TNF alpha [20]. Our patient was treated with NSAIDs and methotrexate and showed good improvement.

## Conclusions

SAPHO syndrome is a rare, frequently underdiagnosed condition. This is due to the different combinations of osteoarticular and cutaneous manifestations that can be present in different models. In our case, medical imaging was very helpful in diagnosing this syndrome, and treatment with NSAIDs and methotrexate was effective. Currently, therapeutic progress has been made, especially with the advent of biotherapies.
